# Intensive Combination Immunotherapy and Neuroinflammation Resolution in a Child With Anti-PCA-1 (Yo) Paraneoplastic Syndrome and 2 Malignancies

**DOI:** 10.1177/2329048X18795546

**Published:** 2018-10-01

**Authors:** Guillermo Philipps, Elizabeth D. Tate, Michael R. Pranzatelli

**Affiliations:** 1 Department of Pediatric Neurology, Golisano Children’s Hospital of Southwest FL, Fort Myers, FL, USA; 2 National Pediatric Myoclonus Center and National Pediatric Neuroinflammation Organization, Inc., Orlando, FL, USA

**Keywords:** PCA-1 syndrome, pediatric neuroinflammatory disorders, adrenocortical carcinoma, Down syndrome, OMS, pediatric paraneoplastic cerebellar degeneration, acute lymphoblastic leukemia, cytosine arabinoside-induced ataxia, ANNA-1 (Hu) syndrome

## Abstract

Paraneoplastic cerebellar degeneration is rare and noteworthy in children. In this 7-year-old, it was documented to have occurred within a year of ataxia presentation. The instigating cancer was stage III adrenal adenocarcinoma, remitted after surgical resection at age 2. When her severe ataxia progressed, neuroinflammation was characterized by high cerebrospinal fluid Purkinje cell cytoplasmic antibody type 1 titers, oligoclonal bands, and neurofilament light chain. The immunotherapy strategy was to replace IV methylprednisolone, which lowered Purkinje cell cytoplasmic antibody type 1 titers without clinical improvement, with induction of adrenocorticotropic hormone/intravenous immunoglobulin/rituximab (ACTH/IVIG/rituximab) combination immunotherapy, ACTH/dexamethasone transition, and intravenous immunoglobulin maintenance. She became self-ambulatory and cerebrospinal fluid inflammatory markers regressed. Down syndrome predisposed her to a second cancer, pre-B acute lymphoblastic leukemia, 4 years later. Despite reversible cytosine arabinoside-provoked cerebellar toxicity, the ataxia is stable on monthly intravenous immunoglobulin without relapse, now 5 years after initial diagnosis. This report illustrates the use of cerebrospinal fluid biomarkers to detect, target, and monitor neuroinflammation, and successful combinations of immunotherapy to better the quality of life.

Purkinje cell autoimmunity is now known to comprise a variety of demonstrable autoantibody disorders. The Purkinje cell cytoplasmic antibody type 1 (PCA-1 or anti-Yo) is an anti-onconeural autoantibody, giving rise to an ataxia-predominant paraneoplastic syndrome in women with breast or gynecologic cancers.^1^ Purkinje cell cytoplasmic antibody type 1 is prone to development of paraneoplastic cerebellar degeneration, which can be the presenting sign of cancer, or a delayed phenomenon in 30%.^2,3^ Studies of the adaptive and innate immune response are few. Immunotherapies, such as steroids, intravenous immunoglobulin (IVIG), and plasma exchange are used with limited success.^1^ With some exceptions,^3^ the prognosis is poor; most adults become bedridden.^1^ We now present observations on the neuroimmunologic profile, and clinical and immunologic responses to a novel treatment approach in a child with Purkinje cell cytoplasmic antibody type 1-induced paraneoplastic cerebellar degeneration.^4^

## Case Overview

Prior to any concerns about neuroinflammatory disorders, the patient carried diagnoses of Down syndrome; stage III adrenocortical carcinoma, which was resected at the age of 2 (along with her left kidney); static mild congenital hydrocephalus (not shunted, stable); reactive airway disease (prn albuterol); and developmental delay (speaking in sentences at 5, toilet trained at 6). The family history was pertinent for multiple sclerosis in the maternal great aunt and gene-loaded for cancer on the paternal side (leukemia)—father deceased from grade IV glioblastoma multiforme—and maternal side (breast cancer). At the age 7 years, she presented with progressive ataxia, which was not recognized as being paraneoplastic until 3 months later. Four years afterward, she developed acute lymphoblastic leukemia, as predisposed by trisomy 21. A p53 mutation, causing risk of recurrent malignancies in keeping with Li Fraumeni syndrome, was discovered. It is noteworthy that the appearance of the paraneoplastic syndrome was delayed 5 years, and the interval between the 2 malignancies spanned 9 years. Additional clinical description and a detailed clinical course summary are provided in Table 1.

**Table 1.** Clinical Course by Clinic Visit.^a^

Clinic Visit	Time After Ataxia (Months)	Drug/Biological Treatments	History and Neurological Examination
1	1	None	New-onset ataxia, progressing. Intermittent horizontal nystagmus, low tone. Not cooperative for proprioception testing. Gait wide based and unsteady. Falls over easily with changes in position. One month prior MRI brain report: enlarged lateral ventricles, unchanged from prior studies. Dx: ataxia of unknown etiology
2	2	IVIG 1 g/kg/d × 2 days	Worsening ataxia needs to hold onto walls. CSF: normal protein/WBC. Normal MRI c/t/l-spine. EMG/NCV normal. Horizontal nystagmus, DTR reduced. Very unsteady gait
3	4	IVIG 1 g/kg/d × 2 days, IV MPRED 30 mg/kg/d × 3 days, PRED taper (1mg/kg/d × 3 days, 0.75 mg/kg/d × 3 days, 0.5 mg/kg/d × 3 days and stopped)	Ataxia persists. Using a walker. No significant improvement. Exam unchanged. Paraneoplastic panel positive for anti-yo (PCA-1) antibodies: serum 1:15360, CSF 1:256. Repeat CSF 1WBC, protein 38.6
4	5	IVIG 1 g/kg/day × 1 day (repeated monthly), IV MPRED 30 mg/kg/d × 1 day (repeated monthly)	Ataxia not clearly progressing anymore. Can walk with hand held. Difficulty using a walker. Repeat PCA-1 titers: Serum 1:1920, CSF 1:128. P53 mutation detected—missense mutation in exon 8
5	6	Monthly IVIG 1 g/kg/d × 1 day, and IV MPRED 30 mg/kg/d × 1 day	Ataxia again progressing. CSF with oligoclonal bands. Exam: highly unsteady with standing. Severe ataxia
6	8	Monthly IVIG 1g/kg/d × 1 day, PO DEX 7 mg/m^2^/d × 3 days, RTX 300 mg/m^2^ × 4 weekly	Ataxia stabilized and improved. Falling less often. Can now throw a ball from standing. On exam, gait wide based, but can bend over, but falls
7	10	Monthly IVIG 1g/kg/d × 1 day. ACTH: 75 IU/m^2^ BID × 1 week, 75 IU/m^2^ daily × 4 weeks, 75 IU/m^2^ QOD × 3 weeks, 65 IU/m^2^ QOD × 1 week, 55 IU/m^2^ QOD × 1 week	Ataxia worsened again 2 months after rituximab completed. Initiated ACTH 4 mo treatment course with improvements. Minor side effects of increased BP. Exam: sways with standing position. Base in gait narrower. Turns and bends over without falling. Repeat MRI brain—cerebellar atrophy
8	12	Monthly IVIG 1 g/kg/d × 1 day, ACTH: 45 IU/m^2^ QOD × 1 week, 35 IU/m^2^ QOD × 1 week, 25 IU/m^2^ QOD × 1 week, 15 IU/m^2^ QOD × 1 week, 10 IU/m^2^ QOD × 1 week, 5 IU/m^2^ QOD × 1 week	Seizure and altered mental status secondary to elevated BP. Started on levetiracetam. BP managed with enalapril. Balance improving. Can pick up things without falling. Climbing up and down stairs. Falls with quick turns, but less often. On exam, cushingoid with weight gain. Mild irritability
9	15	Monthly IVIG 1 g/kg/d × 1 day, PO DEX 7 mg/m^2^/d × 3 days/months	CSF PCA-1 titer: 1:8. Off ACTH for past month. Balance continued to improve. Can run. Intermittent worsening of balance when ill. Off levetiracetam. BP normalized
10	22	Monthly IVIG 1 g/kg/d × 1 day, PO DEX 7 mg/m^2^/d × 3 days/months	Balance stable. Does not sleep well despite melatonin. Per PT report: Can walk 200 to 300 feet without falling (compared to 10 feet, 12 months prior). Mild–moderate unsteadiness with standing on 2 feet. Can stand on one leg with minimal assistance. Stance 10 to 15 inches (was >18)
11	37	Monthly IVIG 1 g/kg/day × 1 day, PO DEX 7 mg/m^2^/d × 2 days/months	Gait stable to improved. Still with intermittent worsening of ataxia when ill. Continued sleeping problems, failed clonidine due to low BP. Exam, stands steadily with minimally wide based stance. Minimal unsteadiness
12	48	Monthly IVIG 1 g/kg/d × 1 day; PO; DEX 7 mg/m^2^/d × 2 days/months. IV MPRED 2 mg/kg/d × 2 days; IV DEX 7 mg/m^2^ × 1 day	Ataxia stable. However, ambulation limited by left hip/knee pain for past 3 months. Left hip effusion found. Suspicion for autoimmune process. Improved with IV steroids
13	51	Monthly IVIG 1 g/kg/d × 1 day, COG AALL 11311	New onset thrombocytopenia during steroid wean. Elevated inflammatory markers. Bone marrow positive for Pre-B ALL. CSF negative for malignancy. ALL in Remission at day 15. Cytogenetics showed near tetraploidy. Heterozygous for TPMT. Repeat PCA-1: serum undetectable, CSF 1:32
14	57	Monthly IVIG 1 g/kg/d × 1 day, chemotherapy	At home, ataxia stable. Developed Altered mental status and worsened ataxia secondary to Ara-c toxicity. ALL in remission. MRI brain unchanged from 2013 study with cerebellar atrophy and ventriculomegaly
15	62	Monthly IVIG 1 g/kg/d × 1 day, maintenance chemotherapy	Recovered from Ara-c toxicity. Mental status back to her normal. Cognition also improving. Ataxia stable. Continues to be able to do stairs. ALL in remission. Exam: child happy and interactive. Fairly steady gait, mild wide base
16	68	Monthly IVIG 1 g/kg/d × 1 day, maintenance chemotherapy	Continues in remission from ALL. Ataxia remains unchanged. Repeat CSF PCA-1 antibody titer 1:8. Exam unchanged from last visit

Abbreviations: ACTH, adrenocorticotropic hormone; ALL, acute lymphoblastic leukemia; Ara-c, cytosine arabinoside; BID, twice a day; BP, blood pressure; COG, Children’s Oncology Group; DEX, dexamethasone; CSF, cerebrospinal fluid; IVIG, intravenous immunoglobulin; MPRED, methylprednisolone; PO, orally; PRED, prednisone; RTX, rituximab; TPMT, thiopurine S-methyltransferase gene; QOD, once a day.

^a^COG AALL 1131: Chemotherapy days 1-14: Ara-C IT 70 mg/m^2^, Vincristine IV 1.5 mg/m^2^/dose days 1 and 8, Methotrexate 15 mg IT day 8, Prednisone 30 mg/m^2^/dose BID PO days 1-14, PEG-Asparaginase IV 2500 units/m2 day 4. Days 15-29: Vincristine IV 1.5 mg/m^2^/dose day 15 and day 22, Prednisone 30 mg/m^2^/dose BID PO days 15-29, Methotrexate 15 mg IT day 29.

## Methods

### Clinical

The patient was referred to the National Pediatric Myoclonus Center for second evaluation of clinical deterioration. Parents gave written informed consent for their child to participate in this institutional review board–approved study of immunological abnormalities in opsoclonus–myoclonus as a related neuroinflammatory/paraneoplastic disorder (SIU SOM, Springfield, Illinois). Clinical data were collected, and extra cerebrospinal fluid and blood for research purposes were obtained from lumbar puncture and venipuncture performed for clinical reasons. Immunotherapy was given in clinical practice, not a drug trial, by the local treating physicians. Given the clinical gravity of paraneoplastic cerebellar degeneration and the persistent ataxia, parents were reconsented for a second lumbar puncture to rule out ongoing neuroinflammation.^5^ Years later, oncologists at the referring hospital performed 2 lumbar punctures to rule out central nervous system leukemic infiltration and subsequent laboratory testing. Western institutional review board (Puyallup, Washington) conferred exempt review status for retrospective data analysis. A modified Opsoclonus Myoclonus Evaluation Scale (nystagmus substituted for opsoclonus) was used to compute Total Score (0-36) from videotapes.^6^ Retrospective gait ataxia scoring by the first author was used to summarize ataxia evaluations from the clinic. The patient’s mother gave written approval for publication of this report with videotapes, including recognizable facial images necessitated by the presence of nystagmus.

### Laboratory

Lymphocyte subsets were measured in cerebrospinal fluid and blood by flow cytometry in the clinical laboratory as previously described.^6^ Lymphocytes gated were 1633 from 14 mL of fresh cerebrospinal fluid from the first visit lumbar puncture and 6838 from 14.5 mL for the second lumbar puncture at the National Pediatric Myoclonus Center. Cerebrospinal fluid oligoclonal bands not found in serum (positive if ≥2) were measured by isoelectric focusing with immunofixation at ARUP Lab (Salt Lake City, Utah).

Cerebrospinal fluid and serum immunobiomarkers were measured in Dr Pranzatelli’s neuroimmunology laboratory by enzyme-linked immunosorbent assay using commercial kits, as per the manufacturer’s instructions. The neurofilament light chain kit was purchased from Ulman Diagnostics (Umeá, Sweden; lot no. 70189); the microglial/macrophage marker soluble chitinase 3-like 1 (sCHI3L1) kit, from R&D systems, Inc (Minneapolis, Minnesota; lot no. 298223). Both were used as previously described.^7,8^

A commercial paraneoplastic serologic evaluation was performed by Mayo Clinic Lab (Rochester, Minnesota) for ANNA Type 1, 2, and 3, anti-glial nuclear antibody type 1, Purkinje cell cytoplasmic antibody (Purkinje cell cytoplasmic antibody type 1, 2, and Tr), amphiphysin antibody, and CRMP-5-immunoglobulin G; antibodies to GAD65 and to N-methyl-D-aspartate (NMDA), γ-aminobutyric acid (GABA), or α-amino-3-hydroxy-5-methyl-4-isoxazoleproprionic acid (AMPA) receptors; SRP, striational, P/Q-Type calcium channel, N-Type calcium channel, ACh receptor binding, AChR ganglionic neuronal, and neuronal (V-G) K+ channel antibodies.

Published control data from Dr Pranzatelli’s neuroimmunology laboratory were obtained from children with noninflammatory neurological disorders and non-neurological disorders who underwent lumbar puncture as part of clinical diagnostic testing. Those data provided medians and reference ranges for use in comparison with the index patient.

## Results

### Clinical and Radiological Response to Immunotherapy

**Figure 1.**
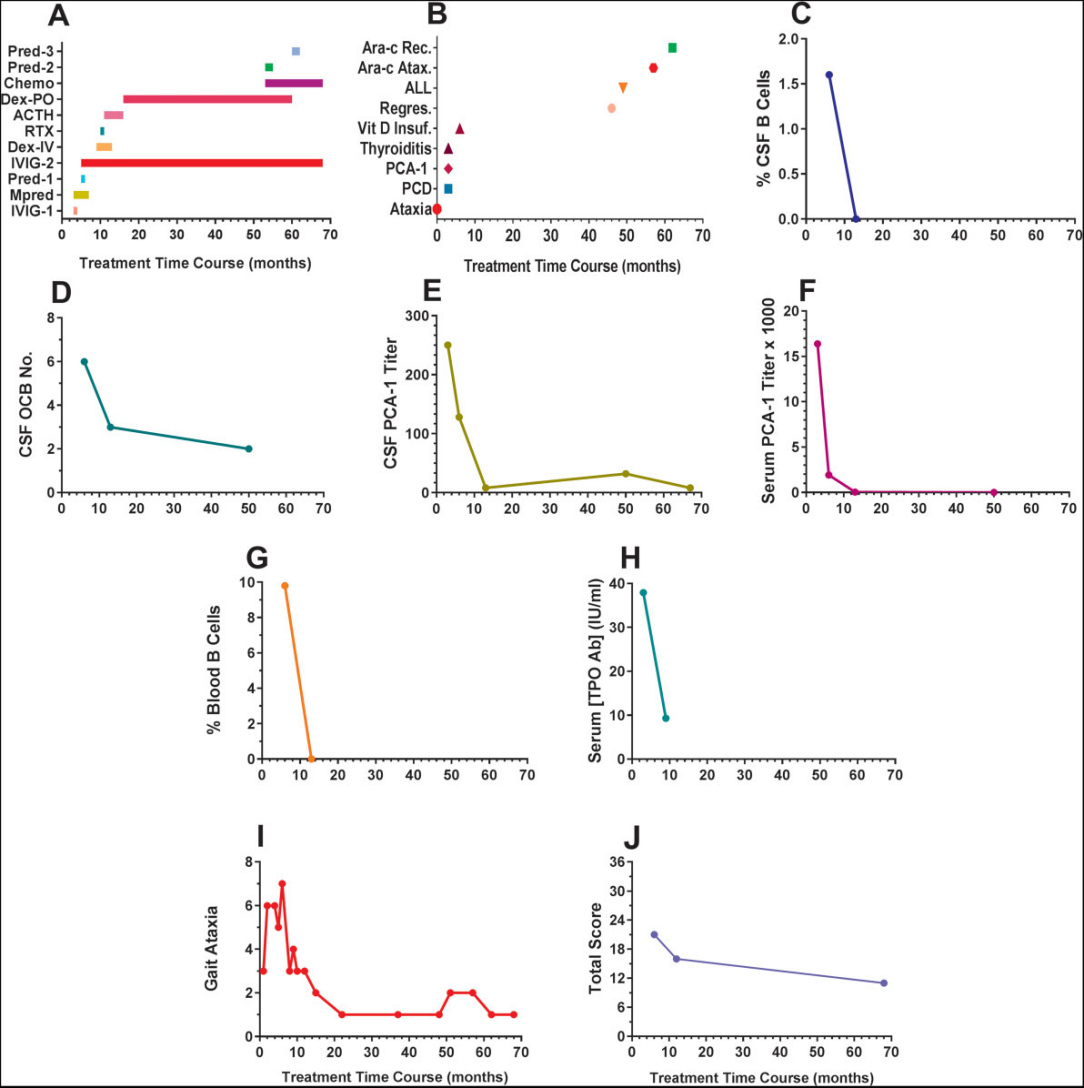
Time course of clinical and CSF measures and effect of immunotherapy. A, Treatment record. Zero marks ataxia presentation. Sequence of treatment reads from bottom to top of Y axis. The −1, −2, −3 suffixes on treatments refer to treatment periods, not individual doses. Intravenous immunoglobulin (IVIG) was monthly; methylprednisolone (MPRED) IV pulse; Adrenocorticotropic hormone (ACTH) twice daily, then daily, then once a day SQ; DEX was 21 mg/m^2^ ÷ TID × 3 pulses, first intravenous, then orally. Treatment in the first 12 months was for induction, then maintenance from 12 to 49 months, then for acute lymphocytic leukemia. More detailed reporting of doses and dosing schedule are provided in Table 1. B, Sequence of diagnoses. C, Percent CSF B cells. The control mean is < 1%. D, CSF OCB count. Per reference lab ≥ 2 bands is positive. E, CSF Purkinje cell cytoplasmic antibody type 1 titers. F, Serum Purkinje cell cytoplasmic antibody type 1 titers. G, Percent blood cells. H, Thyroid peroxidase antibody titers. Normal range is < 9.0 IU/mL. I, Ataxia score. Scale: 0, normal; 1, walks independently, somewhat wide base, steady; 2, walks independently, quite wide base, not falling; 3, walks independently, quite wide base, falling; 4, requires walker, doable; 5, requires walker, difficult; 6, needs to hold on walls to walk; 7, not independently walking; 8, nonambulatory. J, Total Score on a modified Opsoclonus Myoclonus Evaluation Scale. Clinical interpretation is mild severity if score 0 to 12; moderate, 13 to 24; severe, 25 to 36. Ara-cx Rec. indicates cytosine arabinoside recovery; Ara-c Atax., cytosine arabinoside-induced cerebellar ataxia; CSF, cerebrospinal fluid; DEX, dexamethasone; OCB, oligoclonal bands; PCD, paraneoplastic cerebellar degeneration; Regres., regression (mild ataxia 1 week before IVIG was due); Vit D Insuf., vitamin D insufficiency.

The treatment sequence, involving a multiplicity of immunotherapeutic agents, is shown in Figure 1A. In brief, the initial regimen of methylprednisolone and prednisone after only a single treatment of intravenous immunoglobulin was changed substantially for the lack of clinical effectiveness. An ongoing regimen of intravenous immunoglobulin was supplemented by a course of rituximab followed by initiation of adrenocorticotropic hormone (ACTH) after IV dexamethasone, then changed to oral dexamethasone after ACTH weaning and discontinuation. Maintenance intravenous immunoglobulin and oral dexamethasone were extended during the cancer chemotherapy for acute lymphocytic leukemia.

The cumulative sequence of diagnoses is depicted in Figure 1B. Besides the initial diagnoses of ataxia, paraneoplastic cerebellar degeneration, Purkinje cell cytoplasmic antibody type 1, and autoimmune thyroiditis, the patient was subsequently shown to have vitamin D insufficiency and received vitamin D supplementation. At 46 to 50 months before the presentation of acute lymphocytic leukemia, the patient began to experience mild ataxia regression 1 week before intravenous immunoglobulin was due (ie, 3 weeks after dose) on several occasions, which remitted quickly after intravenous immunoglobulin was given. After exposure to cytosine arabinoside, the patient’s ataxia worsened—a known cerebellar toxicity of cytosine arabinoside—but it recovered. Autoimmune thyroiditis was diagnosed on the basis of positive thyroglobulin at 415 IU/mL (normal < 116 IU/mL) and elevated thyroperoxidase antibodies at 37.9 IU/mL (normal < 9.0 IU/mL). The thyroperoxidase antibodies concentration fell on immunotherapy (Figure 1H). She remains on levothyroxine therapy.

The ataxia response is depicted in Figure 1I. By the end of multimodal induction, ataxia severity had declined by 50%, with the functional improvement of beginning to walk independently, though still ataxic. Further improvement occurred despite switching ACTH to dexamethasone. On long-term intravenous immunoglobulin maintenance therapy, ataxia remained at a low level before and after a small rise associated with acute lymphocytic leukemia/cytosine arabinoside.

Using the modified Opsoclonus Myoclonus Evaluation Scale (Figure 1J), Total Score declined by 24% on ACTH/intravenous immunoglobulin/rituximab induction therapy (Supplemental materials, compare Video 1 and Video 2), although remaining in same moderate severity category (13-24 points). Comparison of the neurologic examinations is shown in Table 2. On long-term maintenance therapy, however, Total Score fell into the upper end of the mild range (0-12 points), a 48% drop from the initial score (Supplemental Material, Video 3). As the Opsoclonus Myoclonus Evaluation Scale also evaluates development, Total Score might not be expected to drop much further due to Down syndrome.

**Table 2.** Neurologic Examinations at the NPMC Before and 6 Months After Initiation of Intensive Immunotherapy.

Feature	Visit 1	Visit 2
Cooperative	Mostly	Mostly
Follows commands	With coaching	With coaching
Dysarthria	Moderate	Mild
Sentences	No	3 words
Cranial nerves	Intact	Intact
DTR	Absent	Absent
Ankle clonus	No	No
Extensor plantar	No	No
Block stacking	0 of 8 (right hand)	3 of 8 (right)
	3 of 8 (left)	6 of 8 (left)
Paperclip in bottle	With anchoring	With anchoring
Finger–nose dysmetria	Moderate	Mild
Muscle tone	Decreased	Decreased
Muscle strength	Normal	Normal
Standing^a^	Wide base, backstepping	Wide base
Gait	Wide base, ataxic	Wide base, ataxic
One-foot balance	No	1 second
Hopping	No	No
Falling	Several times	A few times
Able to run	No	No
Ball throwing	0 of 3 tries	1 of 3 times
Ball catching	0 of 3 tries	1 of 3 tries
Getting off floor	4-limb push off	4-limb push off

Abbreviations: NPMC, National Pediatric Myoclonus Center.

^a^ One-foot gap between feet while standing on visit 1.

**Figure 2.**
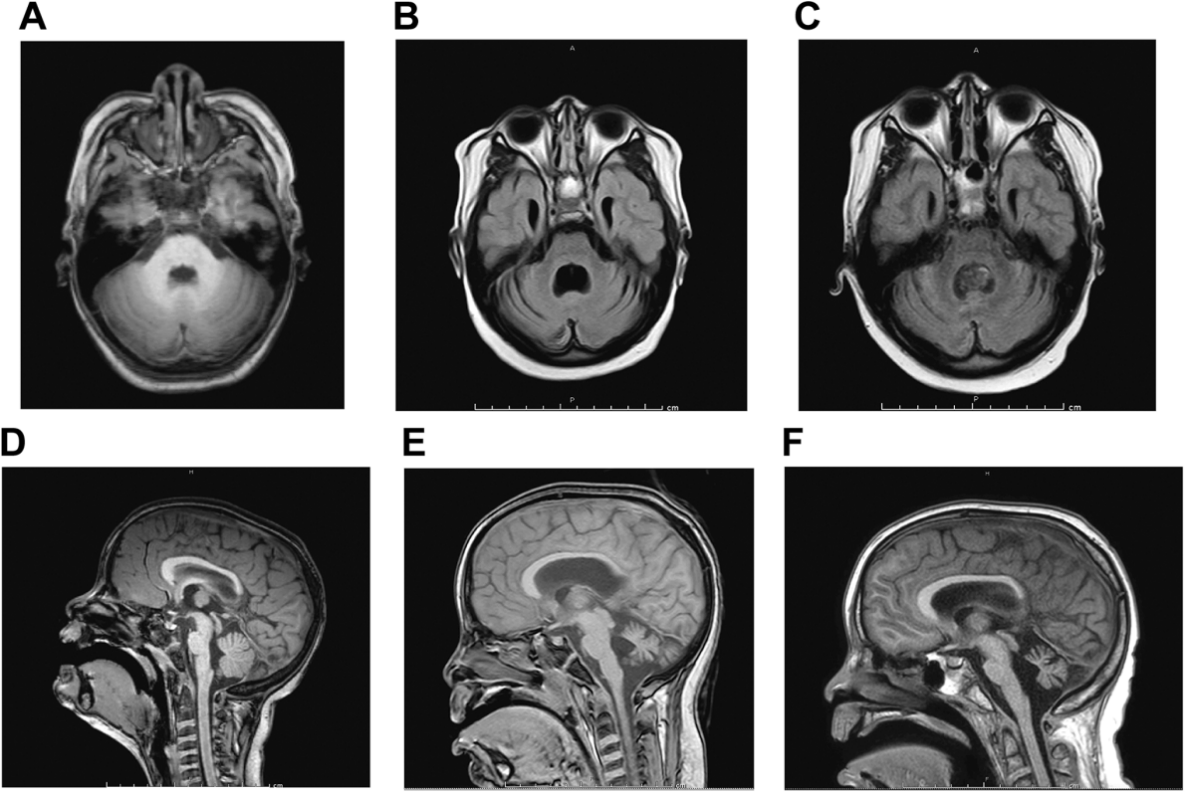
MRI axial and sagittal FLAIR imaging. A, Pre-PCD axial T1 image at time of ataxia onset, 2 months prior to Purkinje cell cytoplasmic antibody type 1 diagnosis (5.5 years ago). Patient had known congenital dilatation of the lateral ventricles, particularly occipital horns. The size of the ventricles remained stable over years with no need for neurosurgical intervention. B, MRI was performed 9 months after onset of symptoms at time of most severe ataxia (5 years ago). It was diagnostic for PCD; T2 image shows cerebellar atrophy with enlargement of fourth ventricle. C, MRI T2 axial image 8 months after acute lymphocytic leukemia diagnosis at time of cytosine arabinoside encephalopathy (1 year ago). Cerebellar volume remained stable when compared to MRI 4 years prior. D, Sagittal image from MRI at onset of ataxia (corresponding to A). Cerebellar vermian volume appears normal. E, On the sagittal image (corresponding to B), there is notable progression of cerebellar vermian atrophy after progression of PCA-1 disease. F, The sagittal image from MRI 1 year ago (corresponding to C) shows no interval change after immunotherapy and systemic chemotherapy. Although only a single image can be shown here, when the entire 2 MRI studies (sagittal, axial, coronal images) were compared with our radiologist, they look remarkably similar, including the dimensions of the vermis and fovea. Despite the severe vermian volume loss, the patient has mild ataxia with minimal impact on the quality of daily life. MRI indicates magnetic resonance imaging; PCA-1, Purkinje cell cytoplasmic antibody type 1; PCD, paraneoplastic cerebellar degeneration.

Neuroimaging of the brain (Figure 2) revealed correlates. The magnetic resonance imaging (MRI) that was obtained 2 months prior to Purkinje cell cytoplasmic antibody type 1 diagnosis (about 2 weeks into the ataxia symptoms) did not show paraneoplastic cerebellar degeneration (Figure 2A and D). Presumably, the cerebellum was normal because it was early in the disease course. How fast the degeneration developed thereafter is unknown, except to say that it had occurred by the next MRI 10 months after the initial scan (Figure 2B and E). The radiologic appearance of paraneoplastic cerebellar degeneration did not improve on immunotherapy nor did it progress (Figure 2C and F).

### Neuroimmunologic Studies

At the first diagnostic evaluation, the main neuroimmunologic observations were mildly increased the frequency of cerebrospinal fluid B cells (1.6%), positive oligoclonal bands (6), and elevated Purkinje cell cytoplasmic antibody type 1 titer (Figure 1 C-E). Cerebrospinal fluid immunoglobulin G index, immunoglobulin G synthesis rate, immunoglobulin G/albumin ratio, protein, and glucose were normal. There was no cerebrospinal fluid pleocytosis (WBC 1/cu mm; RBC 0). The cerebrospinal fluid leukocyte differential was 80% lymphocytes and 20% monocytes. The cerebrospinal fluid CD4/CD8 T-cell ratio was low at 1.8 (normal 2.5-3). The neuronal/axonal marker neurofilament light chain was elevated 10-fold in cerebrospinal fluid at 3254 ng/mL (controls, 300 ng/mL) and also in serum at 722 ng/mL (controls, 22 ng/mL), and the neurofilament light chain cerebrospinal fluid: serum ratio was 4.5. The serum Purkinje cell cytoplasmic antibody type 1 titer was grossly elevated at 1:15360 (Figure 1F). The blood B cell frequency of 9.8% was not elevated (Figure 1G).

At 7 months on intensified combination immunotherapy, there was amelioration of adaptive immunity: undetectable cerebrospinal fluid B cells, and reduction in oligoclonal bands count (3) and Purkinje cell cytoplasmic antibody type 1 titers (1:8). WBC count was 4/cu mm (81% lymphocytes/19% monocytes); RBC 0. Cerebrospinal fluid immunoglobulin G index was normal; immunoglobulin G index, slightly elevated at 0.71. The cerebrospinal fluid CD4/CD8 T-cell ratio normalized. The frequency of γδ T-cells, primarily considered to be innate immune cells, was normal at 2.6% in cerebrospinal fluid and 10.8% in blood. The concentration of the M2 macrophage marker CHI3L1 in serum was 36 ng/mL at the low range of controls (431 ng/mL). Blood B cell frequency was 0%.

When acute lymphocytic leukemia presented (on prednisone 15 mg/d and intravenous immunoglobulin 8 days prior), cerebrospinal fluid oligoclonal bands was 2, immunoglobulin G synthesis rate 11.5 mg/d (vs < 8), cerebrospinal fluid immunoglobulin G 8.3 mg/dL (vs ≤ 6), and albumin index (10.9 vs ≤ 9). These were interpreted as mild elevations.

## Discussion

This case introduces several novelties. First, intensified combination immunotherapy induced marked clinical improvement, whereas prior methylprednisolone/intravenous immunoglobulin also reduced Purkinje cell cytoplasmic antibody type 1 titers but with little clinical benefit. Second, signs of neuroinflammation steadily declined, all but a few normalizing, corresponding to functional improvement. Third, no major relapses occurred on dexamethasone/intravenous immunoglobulin maintenance therapy, despite Down syndrome–associated leukemia and chemotherapy. Fourth, the features are consistent with adult-onset Purkinje cell cytoplasmic antibody type 1, but with better outcome. Fifth, cerebrospinal fluid neurofilament light chain was elevated, as in opsoclonus–myoclonus syndrome and anti-ANNA-1 (Hu) syndrome, suggesting a shared immunopathologic aspect of otherwise distinct clinical syndromes. Sixth, the patient had concomitant autoimmune thyroiditis, which responded to the same immunotherapy given for the paraneoplastic syndrome. Seventh, the patient was vitamin D insufficient, a common association with autoimmune disorders. Eight, there were two malignancies: the first, rare and previously not known to be associated with paraneoplastic cerebellar degeneration, the second, associated with Down syndrome.

The “Time is Brain” motto has been applied to the cerebellum: “Time is cerebellum.”^9^ Despite the cerebellum’s capacity for compensating and restoring lost functions, the therapeutic opportunity for intervention occurs early in patients with cerebellar diseases, particularly immune ataxias. Advanced cell loss degrades cerebellar “reserve,” hastening the transition from a restorable or treatable state to an untreatable one.^9^ Timely immunotherapy is necessary to treat neuroinflammation comprised of the adaptive immune response of B cells and T cells, among others, and possible involvement of the innate immune system, which is involved in neurodegeneration. Yo-paraneoplastic cerebellar degeneration tumors are infiltrated by large numbers of B and T cells, some organized in tertiary lymphoid structures, and Yo-paraneoplastic cerebellar degeneration-manifesting ovarian carcinomas harbor at least 1 genetic alteration of Yo-antigens thought to trigger the breakdown of immune tolerance.^10^ There is a differential genetic susceptibility to anti-Yo per cancer with primary human lymphocyte antigens class II involvement.^11^

The combination of ACTH, intravenous immunoglobulin, and rituximab, with or without transitioning to dexamethasone for maintenance prior to weaning, has been successful in children with opsoclonus–myoclonus syndrome, both clinically and against neuroinflammation.^6^ Rituximab (anti-CD20) targets B cells. In a pilot study of rituximab in 9 adult patients (anti-Yo and anti-Hu), 3 patients improved ≥ 1 Rankins Scale point after monthly IV rituximab 375 mg/m^2^.^12^ Combination immunotherapy with dexamethasone, intravenous immunoglobulin, and rituximab (DEXIR-CI) also has similar effects.^13^ Rituximab influences neuroinflammation whether or not the clinical benefit occurs during the 4-week infusions or thereafter. In the broader view, there are now several different treatment protocols for paraneoplastic disorders available to child neurologists^13-16^ so clinical deterioration, partial response, and relapse can be addressed by forward-thinking, biomarker-assisted initial treatment planning or mid-course corrections.

In comparison of our case with the Purkinje cell cytoplasmic antibody type 1 syndrome in adults, early cerebrospinal fluid studies in adults reveal lymphocytic pleocytosis, oligoclonal bands, and elevated protein,^1^ but our patient did not have pleocytosis or elevated protein. The B-cell frequency was lower than usually found in OMS. As with our case, anti-Purkinje cell cytoplasmic antibody type 1 antibodies may persist for years.^2^ Serum Purkinje cell cytoplasmic antibody type 1 has recently been reported outside the context of cancer in 77% of children with attention deficit hyperactivity disorder (ADHD) and 22% of controls in association with elevated interleukin 6 (IL-6) and IL-10 serum concentrations.^17^ Pending replication of that finding by other investigators, the clinical significance, and presence of any such parallels in adults remains uncertain.

The striking elevation of cerebrospinal fluid neurofilament light chain concentration in pediatric-onset paraneoplastic neurological disorders is significant. An elevated cerebrospinal fluid to serum neurofilament light chain ratio indicates intrathecal concentration. Now described in pediatric-onset ANNA-1 (Hu) paraneoplastic syndrome^18^ and OMS,^7^ it is a useful biochemical measure. Well known to have utility in assessing neuroinflammation in multiple sclerosis and related disorders in adults, the opportunity to utilize it presents itself to child neurologists.

Cytarabine-induced ataxia due to toxicity, which degrades cytoskeleton components like neurofilament,^19^ has been well described.^20^ The antineoplastic and immunosuppressant drug acts through inhibition of DNA polymerase. With intrathecal administration, cerebrospinal fluid cytarabine levels decline with a half-life of 2 hours. The incidence of cerebellar ataxia is up to 14%.^21^ In a study of 418 patients aged 2 to 74 years with leukemia or lymphoma, 8% developed severe cytarabine-induced cerebellar toxicity, especially if > 50 years old, regardless of gender, diagnosis, or regimen.^22^ Drug dose and schedule, cumulative dose, renal and hepatic dysfunction, and use of neurotropic antiemetic drugs can also affect risk.^21^ In our patient, it is possible that paraneoplastic cerebellar degeneration may have made the patient more vulnerable to the cerebellar toxicity, which was clinically reversible; however, the cerebellum, with its extended postnatal development, is particularly sensitive to toxic agents, even in patients without paraneoplastic cerebellar degeneration.

Cerebellar abnormalities associated with Down syndrome and a Down syndrome murine model includes reduced cerebellar volume and granule cells.^23^ Although they may contribute to the hypotonia of Down syndrome, as exhibited by this patient, they do not account for her substantial cerebellar/vermian atrophy or the relatively short course of its development. Pre-paraneoplastic cerebellar degeneration/Purkinje cell cytoplasmic antibody type 1 neuroimaging did not display cerebellar hypoplasia or appreciable atrophy.

Whereas only 0.2% of all pediatric malignant cancers are adrenocortical tumors,^24^ children with Down syndrome are well known to have a greatly increased risk of leukemias.^25^ For acute lymphocytic leukemia, their event-free and overall survival is poorer than in non-Down syndrome acute lymphocytic leukemia.^25^ Although the 5-year event-free survival for adrenocortical tumors is only 54%,^24^ our patient did well. The outcome is better with localized tumors of small volume.^26^ The patient thus far is responding well to chemotherapy. Paraneoplastic cerebellar degeneration also occurs rarely with other pediatric-onset cancers, such as Hodgkin disease.^27^

This patient’s autoimmune thyroiditis, found on screening, presented differently than acute “steroid-responsive encephalopathy associated with autoimmune thyroiditis” (alias Hashimoto encephalopathy),^28,29^ with its requirement for negative brain neuroimaging and exclusion of all other causes. Here, the autoimmune thyroiditis most likely reflects the increased risk of Hashimoto thyroiditis and Grave disease in Down syndrome,^30^ and of having subsequent autoimmune disorders in addition to the initial one in children with perturbed autoimmunity. The thyroiditis was responsive to immunotherapy and has not returned.

Vitamin D insufficiency or deficiency is more common in patients with autoimmune disorders and has been associated with multiple sclerosis, systemic lupus erythematosus, polymyositis and dermatomyositis, rheumatoid arthritis, Behçet disease, type 1 diabetes mellitus, and systemic scleroderma.^31^ Besides autoimmune disorders, the inestimable consequences of vitamin deficiency, which is now a pandemic, include cancers, infectious disease, cardiovascular diseases, and childhood dental caries and periodontitis.^32^ Testing for 25-OH-vitamin D levels and supplementing with vitamin D if low is highly recommended in patients with autoimmune disorders.^31^

## Conclusion

The clinical importance of our observations is that intensive, long-term, biomarker-based, combination immunotherapy made a difference in this rare child with paraneoplastic cerebellar degeneration and anti-Purkinje cell cytoplasmic antibody type 1 paraneoplastic syndrome, both in quality of life and amelioration of neuroinflammation. Given the better outcome than would be predicted from poor treatment responses in adults, this immunotherapy approach should be pursued in others who share the diagnosis. Purkinje cell cytoplasmic antibody type 1 antibodies should be measured in children with ataxia or paraneoplastic cerebellar degeneration presentation, even if years after cancer treatment. Alertness to the increased risk of a second autoimmune disorder and/or malignancy and testing for vitamin D deficiency/insufficiency should be routine. Such patients need long-term follow-up to stay the course.

## Supplementary Material

Supplementary material

Supplementary material

Supplementary material
